# Supplementary material for: Beliefs and Use of Palpatory Findings in Osteopathic Clinical Practice: A Qualitative Descriptive Study among Italian Osteopaths
Source: Healthcare (Basel). 2022 Aug 29;10(9):1647. doi: 10.3390/healthcare10091647 (PMC9498689; doi:10.3390/healthcare10091647)
Supplement: Supplementary file 1 [file healthcare-10-01647-s001.zip › Quotes S1.pdf]

Explanatory quotes of the qualitative research carried out.

S3.1.1. Osteopathic Identity

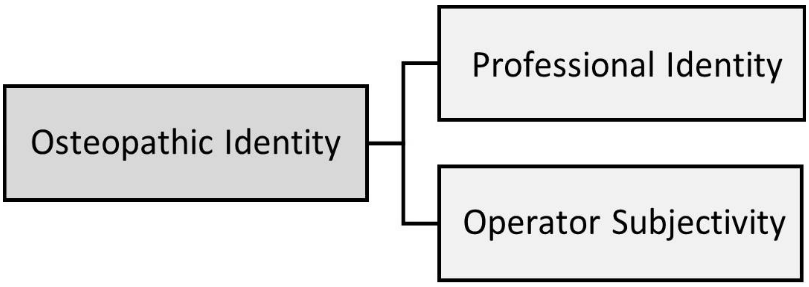

Figure S1. Categories grouped by theme ‘Osteopathic Identity’.

S3.1.1.1. Professional Identity

CF: “... osteopathic treatment without perception doesn’t exist! … our profession is based on touch and touch implies perception.”
BG: “… the hand, how important is the hand? I think that for osteopaths the hand is fundamental so the idea of abandoning something of the profession, I don’t know what it could be but certainly not the palpatory aspect, I think this is very important.”
VL: “… we can’t lose palpation. Actually, I think we have already lost so many things, I wouldn’t leave anything to other professions, we already gave away a lot.”
BL: “… our sensor (the hand) becomes finer in time allowing us to assess small movements and this is our peculiarity. […] a manual therapy that considers only biomechanics or a certain kind of movement or palpation […] is common with other manual therapies. What do we have that is just ours? […] the touch, the perception, how I analyse it […] that is something others don’t have.”

PG: “… maybe it would be important to weigh, give a weight to this main theme of palpation […] we have to find a tool that allows us to characterise our profession, that is univocal. Surely the aspect of perception does characterise it but it is very unreliable and I would therefore put it on a second level, I wouldn’t throw it away but I don’t think it is of primary importance.”

BD: “... a perceiving listening, which is the ability to listen to the tissue but it is actually a listening to the person in general …”
BL: “... of a sort of listening that is less technical but more of a listening to what the patient is giving me …”
BG: “... like a palpatory observation of the characteristics of the tissue …”
CF: “... the perceiving touch, potentially is something we can include within a sensation or a

somatosensory analysis of what we perceive with touch."

CG: "... this osteopathic jargon for which we use "to listen to" that is evidently a synesthesia because one doesn't listen with one's hands, I listen with my ears [...] when we tend to use a figure of speech to say something because we are not able to define it differently [...] it empowers the fact that we are all confused."
LC: "... actually it is an Englishism in the sense that some authors use a metaphoric language 'to listen, listening, general listening or local listening' [...] that also means to consider, to pay attention [...] we are talking about perception which is in the domain of touch, the communicative aspect in the touch."
DFF: "... on this figure of speech I think we should surrender. Both for expressions such as 'perceptual listening', but also 'perceptual observation' or whatever [...] the key word is 'perception'."

S3.1.1.2. Operator subjectivity

CF: "… the operator creates perceptual maps that are the synthesis of what the operator is feeling in that moment, or thinks he or she is feeling in that moment plus the history of the operator […] In function of the operators' experience, in function of the educational background of the operator; the practical experience, so with a hand-on approach of that area but also in treating in general but also theoretical."
BA: "… the importance of the background linked with the educational aspect ..."
BD: "… surely related to the educational curricula of the person but not necessarily those that are the technical competencies but what is really the background of that person, intended like the journey of that person at a 360° […] the journey of personal growth … as a human being."
DFF: "… we can surely have a technical toolbox that we acquire during our educational path, we can train it with different schemes […] then there's this big slice made up by the 'Self/I'; the 'I' operator with its kind of experiences not only technical osteopathic, but of all kinds."

CF: "… depending on how the operator is interacting with the area from a cognitive point of view, meaning if he is concentrated, if he is paying attention or not paying attention, if there are elements that tend to influence him, if he is distracted or not distracted. How many patients he visited, the context in which he is, how he is feeling."
BD: "… it considers all of what the patient told me before … all of what the patient talked about from the moment he/she entered the office ..."
LC: "There's a Self who today is more tired, less tired, more punctual, less punctual, more intuitive, less intuitive, more cognitive, less cognitive."

CF: "… the culture of the operator …"
DFF: "… even cultural aspects […] there is a cultural aspect, the palpation of an Italian osteopath could differ, even just for this aspect, from the perception of a Anglosassone, Indian, Australian, Native American."

DFF: “... from the moment we enter the sphere of perception of a person, we open thousands of windows even the unthinkable that make the person unique therefore the elaboration that is driven by the touch becomes unique …”
CG: “... we put together a whole series of things for which we nearly have a mathematical certainty that a perception can be biased […] we know that our perception by itself is biased.”
DFF: “... my ability to interact with the tissue and to try and have an idea is totally subjective …”
BD: “... I realise that from the moment in which I welcome a person in their totality this could condition the perception because it’s a perception that considers not only the tissue but also to who the tissue belongs to therefore, the lack of objectivity.”

S3.2.1 Evaluation

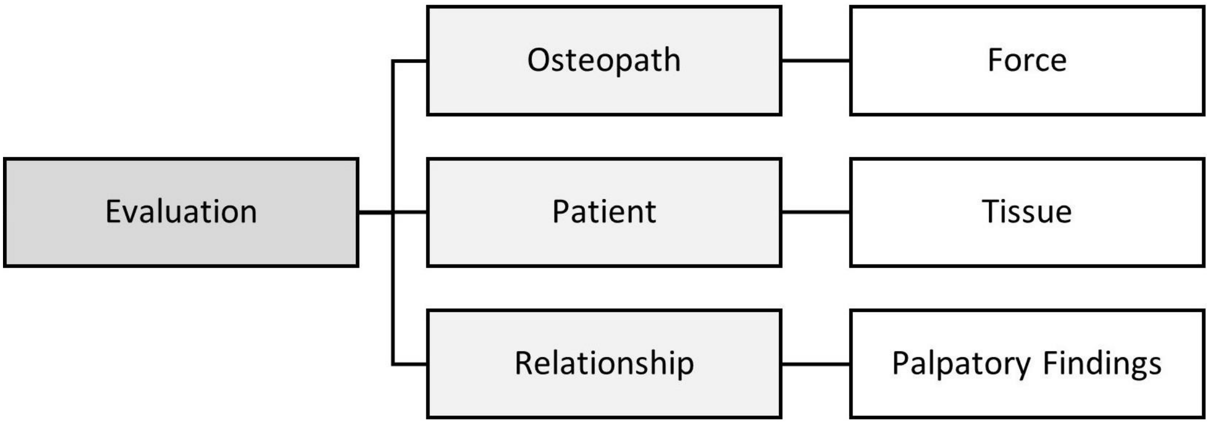

Figure S2. Categories and subcategories grouped by theme ‘Evaluation’.

S3.2.1.1. Osteopath

CF: “... if we look at it from the operator component it means that the perceptual information and therefore of the touch that arrives at the higher centres of the operator's brain tend to take two paths and this is physiology [...] The two then integrate within the operator's brain which in turn creates perceptual maps that are the synthesis of what the operator is feeling at that moment or thinks he feels at that moment plus the operator's history.”
DFF: ”We can say everything we want, that 'self/I' -operator- part has a fundamental weight, despite the technical aspects and surely there must also be the relationship with the patient.”

S3.2.1.1.1. Force

BG: "... about how we use palpation or the hand in daily practice, I can tell you what my experience is, which is basically based on the use of an extrinsic force on the tissue. A force that is dosed according to what it is the request you want to make to the tissue …"
BA: "... I use my hand to understand which forces to use."
CF: "The use of touch to understand what are the reactions of the tissue in relation to the specificity of a potential diagnosis. Therefore, how the tissue reacts in function to specific stresses that the operator induces in that specific area to understand how the tissue responds."

DFF: "'Forces, forces at play' right? An aspect that is part of our evaluation phase [...] but then that guides our entire therapeutic practice …"
DFF: "'I touch, induce, activate the flexors' or 'move', in short, we understand, as we already knew in reality, that we (osteopathic community) do many things, often very different [...] there is who conceives the patient's evaluative sphere in a completely different way and act, from a therapeutic point of view, with other tools, in short this creates a 'gap', a 'canyon', let's call it what we want, that should make us reflect for a moment."
BA: "So listening is not a gesture, a passive reality, that is [...] but I give information, a small, large input, etc., a force, the patient's reaction is what guides me in the evaluation, in the information that is useful to then decide to do something."

BG: "... the use of an extrinsic force on the tissue, a force that is dosed according to what is then the, let's say, the request you want to make to this tissue, i.e. if you want to simply evaluate an aspect of consistency, an aspect of temperature or if you want to evaluate an aspect of motility and if you want to go for a motility evaluation, the extrinsic force that I use is an isometric contraction of the deep flexor muscles of the hand."
BL: "... it is the sensitivity of the sensor that refines the capabilities of investigation and inspection. [...] Both in the methods of approach: that is, at the beginning the student is taught the flexion of the deep hand flexors to get in depth but then from the moment you understand that your afferential hand sensor is refined, you go to explore also in another way. So there is a basic model for building what a process is, so let's say that it must be uniform, in the sense that we must all probably look for."
CF: "In general it depends on the type of touch that one wants to use, but basically what I use is actually a force so it is a pressure that the operator induces at different levels, we want to call them Newton, at different Newtons, different pressures with a different force on the various parts of the body."
BA: *"I squeeze something, or pull something ..."*

S3.2.1.2. Patient

CG: "... we are used to being focused on what we do, we are rightly osteopaths, but I find it difficult to have a palpatory finding regardless of the fact that there is a patient …"
LC: "... the insertion of the patient in the process is a step that we must consider …"
CG: "... 50% is what we touch and the other 50% is the patient who is touched …"
CG: "... we define ourselves as medicine aimed at the patient, a patient-centred therapy."

CG: "... the patient responds with tissue, it is a tissue response…"
CF: "... the contact with the patient that occurs through the positioning of the hand on the patient's body area generates in turn a contact, therefore skin-to-skin, which in turn generates information for the patient himself and information for the operator ..."

S3.2.1.2.1. Tissue

CF: "... identify one of the salient characteristics of the tissue that trigger a decision-making mechanism on what the operator is perceiving …"
CG: "The rationale for this type of approach is basically when "I like-dislike" an area, and it becomes interesting from a clinical point of view [..] from a palpatory point of view then the tissue responds to me so it has a reactivity capacity [...] then my hand tells me this interests me now …"
CF: "... the reactivity of the tissue which is a function of a series of biological rhythms ..."

BA: "… movement as an important variable in the evaluation we make and I speak of a movement that has to do with very small ranges [...] what I personally use cannot ignore, in my opinion, the movement."
CF: "Personally I believe that the quality of the tissue and therefore the response of the tissue -if we want to remember the TART, if you like it-. The tissue texture alteration, I believe that it can be the key element that triggers this reactivity on the tissue. On the other hand, anything that I personally happen to feel - as I was taught- is the reactivity of the tissue as the main element …"
CF: "... tissue condition, therefore the response of all those characteristics of the touched tissue, as a function of resistance, elasticity, consistency."
BG: "... the question you want to ask the tissue, if you simply want to evaluate an aspect of consistency, temperature or if you want to evaluate an aspect of motility …"
VL: "... the impaired function [...] I consider it, let's say, an adaptation of the system to something that is required of it; and I evaluate this through movement."

BA: "... the concept of tissue consistency, which is always registered through pressure, so as CF said, we speak of Newton, of kg weight, that is, I squeeze something, or pull something ... actually I, for me we are always talking about movement."
PG: "I rely heavily on movement, on the provocation of the symptom and then in sequence also on the aspect of tissue density. So this is what I do a little in clinical practice."
TM: "... following a logical pathway but in a more macroscopic context [...] I first evaluate the movement and then I entrust myself to the palpation; or at the same time through the palpation I also evaluate the movement."

CG: "... responsivity of the patient which is partly what CF says because the patient responds with tissue, it is a tissue response, call it thixotropic, call it whatever you like ..."
VL: "We see osteopathic diagnosis [...] listening to BG and CF in a slightly different way; in the sense that for me palpation is, what I evaluate with palpation, it is not so much a question linked to pressure but to the movement. I feel it is important to analyse the movement, if we put it in simple terms, of the micro movements or -as you know, we have described it- a movement in the neutral zone. So for me, palpation is first of all analysing how the system moves."

S3.2.1.3. Relationship

LC: "... is the relationship with the patient or if you want the patient, maybe I prefer the term 'relationship', then in the shared decision-making process that is defined as the use of palpation."
BD: "So listening to the other, who is not only through words but above all towards what the tissue expresses and what I do in my clinical practice, responds precisely to this: being able to combine what is 'welcoming' a person and therefore 'taking care' of the other in listening and then evaluating through that, which is a routine because I apply a routine."
CG: "... we no longer even talk about person-based medicine but relationship-centred medicine. Therefore, it is no longer an operator centric, it is no longer patient centric, it is a relationship centric. And this here is very interesting, it is very interesting because the patient's responses, the patient's sensations, I use in the treatment.

BA: "If you like the touch, if you like it too much ... how should I change my touch but the dialogue tends to be manual, non-verbal .... [...] therefore the verbal dialogue yes but the 'dialogue' if I had to put it on the scales, it's definitely more manual than verbal … [...] I speak but with my hands.
DAG: "The relationship is present within the question that I imagined myself asking the patient, if I were there as an operator: "what do you feel?" So, in any case the patient is there quietly, both palpatorially and verbally."
PG: "... the provocation of pain, so in a sense having feedback from the person, then establishing a relationship and hearing what the person is saying to me, ok? About what I'm doing."
CG: "... my hand tells me 'this interests you now!' I'm only interested if the patient is also interested… so I ask the patient for feedback, if he has an altered sensation from a palpatory point of view which can be of a different nature …"

CG: "So, I don't know, the thing that I would like to add in an important way is precisely this active and not passive presence of the patient during the whole process, which can be both the evaluation and the therapeutic one afterwards."
DAG: "... what I have begun to include in my clinical practice in recent years is the patient's perspective, which is the subjectivity that osteopathy then already has also in the TART criteria with T, with tenderness. But the patient's perspective has become a little more important."

S3.2.1.3.1. Palpatory Findings

BD: "... listening to the tissue, but in reality what the tissue conveys."
BG: "... a more local aspect, we can define it as a palpatory observation of the characteristics and properties of a tissue in that case therefore taking into account the consistency, density, presence of resistance, barriers in this sense we speak of perceptive listening."
VL: "... the impaired function [...] I consider it, let's say, an adaptation of the system to something that is required of it; and I evaluate this through movement."
LC: "When we define somatic dysfunction is it a palpatory finding or is there something else in it? I often think that [...] there is a problem of definition; then if we have not agreed on a definition we are talking about different things; [...] does palpatory finding allow you to identify an alteration of a function [..]? that is, can we say how through palpation [..] we can define an alteration of a function? Perhaps the palpatory finding is an ok piece, then all the elements of contemporary biomechanics are welcome, so rigidity as CF said, elasticity, now I don't remember all those he listed …"

S3.3.1. Osteopathic Diagnosis

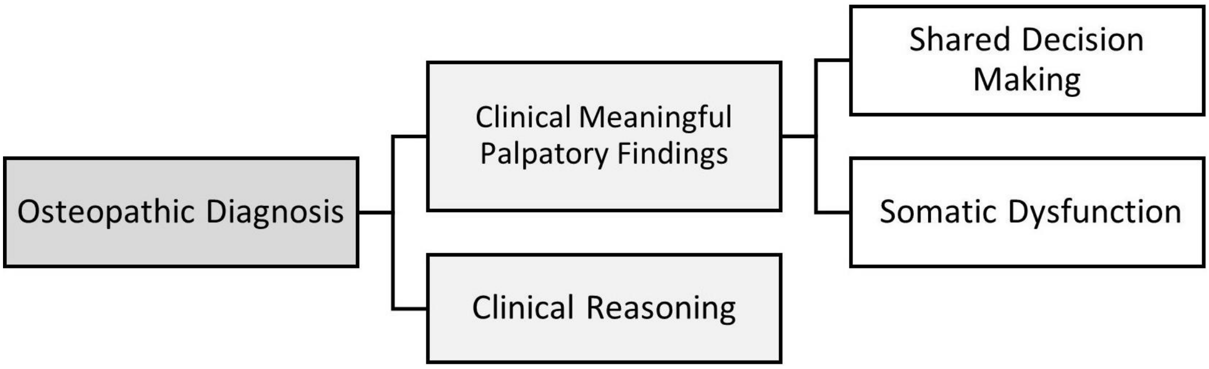

Figure S3. Categories and subcategories grouped by theme 'Osteopathic Diagnosis'.

CG: "... we are talking about the assessment, but since we are in a complex system, the relationships between elements can be observed only retrospectively [...] I doubt we have the possibility to verify if our decisional process is coherent or not [...] trying to find direct links between cause and effect is a waste of time."
LC: "... when we talk about a complex phenomenon, it is very difficult to find just one test [...] there has to be a series of elements we put in. One day we will have to decide how as well as which (elements) we should use."
TM: "Subsequently, after I have finished, I have exhausted all my osteopathic evaluation that makes use of palpation, I put in relation all the different dysfunctions that I have managed to bring out, subsequently, let's say, puts them in relation with the reason for consultation and testing to make a synthesis that is imbued with clinical reasoning and is based on my knowledge, experience and then I decide if necessary what and what to treat [...] palpation, possibly if I find some tissue alterations I test the movement and then memorise and then subsequently compare I evaluate what I find, I reason and I make a synthesis to understand what the most suitable therapeutic approach may be".

BG: "...There is a specific diagnostic moment that then needs to be amplified, or completed, by a much greater assessment or relationship with the patient to make a real and proper Osteopathic Diagnosis."
LC: "Another bias of mine is "considering osteopathy centred on people", and if it is centred on the person, the decision-making process is shared. And in a shared decision-making process, if I touch a thing and decide how to treat it myself ... non-verbally ... that is, because that's another gap, that is, I touch, and then I want verbal feedback ... then we have to decide. It is probably my cognitive bias but it is written all over the place, which is centred on the person, and today we say "about the relationship". So I start from this assumption …"

LC: " … inserting the patient in the process is something we need to consider …"
CG: "We know our perception alone is biased is the thing that makes us want to combine it with someone else's perception of this thing."
CG: "Let's imagine that this here is the set of my perceptions, and inside there is a certain number of biases. Now if I also take the patient's perceptions ... this subset (the intersection) is here like a Venn's diagram, right? When I see this intersection here I have a certain probability [...] there is a certain possibility that a certain number of biases remain closed out of the central set, right ?"

BA: "So listening is not a gesture, a passive reality, that is […] but I give information, a small, large input, etc., a force, the patient's reaction is what guides me in the evaluation, in the information that is useful to then decide to do something."
TM: "... I (osteopath) relate all the different dysfunctions that I have been able to bring to light, then I relate them to the reason for the consultation and try to make a synthesis that is steeped in clinical reasoning and is based on my knowledge, experience and then I decide which and what to treat ..."
LC: "... in a shared decision making process to have process addressers which are based on a verbal feedback from the patient just considering the body awareness of the patient which can be labile, that is it can be more or less active, we need other process addressers which can be subjective as well, [...] in my opinion linked to the disorder, linked to the perception, to the sickness, to the experience of the disorder or of something he or she considers related to the disorder and when we go to the comparable symptoms it is also far away."

S3.3.1.1. Clinically Meaningful Palpatory Findings

LC: "... If, on the other hand, I do as it is written on the osteopathic guidelines, the only ones we have, I check and then treat or not and therefore I am saying, the neutral zone is fine, the present movement is fine, ok, but it does not necessarily mean that what I have perceived has a meaning, I try to verify it, since it is not absolute ok, and then I treat or not."
BG: "... that the somatic dysfunction has its clinical component, its semeiological component, is out of the question in my opinion."
LC: "… who may or may not have it (referring to clinical role of SD) ... in fact we speak of "severity" of the dysfunction, of "clinical relevance" of the dysfunction."
LC: "Well, it seems to me that we have treated something that we ONLY palpate as a fever at 42! That is, as something that has to be treated because it has a meaning. We don't know if it has a meaning!"

S3.3.1.1.1. Shared decision making

CG: "... osteopaths have the "magnet" for discomfort saying that their hands found places that patients did not know they had as areas that were active and interesting from a somatic point of view and they discovered thanks to palpation that they were interesting from a somatic point of view and so in my opinion one of the possible roles of palpation is just this, to bring back on conscious levels of the patient some areas of the body that at that point, ... he can attribute or she can attribute a meaning."
LC: "… in the shared decision-making process the patient has a part because he or she checks if what I am touching has an impact on his body functions, which he or she recognises with his or her body awareness ... the 'familiar symptoms', the patient is aware of them, he or she tells you; the 'signs of comparison' are those where the osteopath accesses through an intuition and a palpation to some aspects that are not accessible to the body awareness of the patient in that moment and therefore the verbal feedback arrives through the mediation of the palpation of the osteopath."
DAG: "... Then about the role of the patient ... for example we can make the patient aware of an area that he or she may not consider …"

S3.3.1.1.2. Somatic Dysfunction

LC: "… somatic dysfunction is a compromised function and an altered function, and in osteopathy we also describe what these functions are and that it is related [...] to a body framework, therefore to the soma ... a dysfunction is not a dys-structure, it is a related alteration of function emerging in a body region or pattern. … It's not an altered soma related to an altered function, it's an altered function …"

BA: "To understand whether this term actually exists in the clinic, which reports the altered function of body systems, where, in my opinion, it can be placed in the context of adaptation ..."

BG: "... when making a diagnosis we generally have to consider a clinical aspect, a semeiological aspect, an aspect of pathophysiology [...] The moment we are talking about altered function, we are talking about a clinical aspect."

LC: "... it seems that this entity that we (osteopaths) palpate represents something in the soma to convey the effects ... related to the relationship between operator, patient and environment."

BA: "... I agree that if you talk about function, the word itself says it, the challenge is to understand what function this is [...] So, in the whole, as we know, in osteopathy we talk about health, so it expresses something really macro, gigantic, enormous, made up of every little piece, but this is the function of the whole. 'Somatic', for me, is perhaps meant to convey a message of the whole ..."

DAG: "Somatic dysfunction, which was already for me a gateway to the patient system."

LC: "... it is to be placed alongside something that relates to the person's health processes and that we use to analyse treatment … that stuff is related to a general allostatic load ..."

VL: "... when I palpate the dysfunctional part [...] for me it may not be related to the problem but it may be very much related to the adaptation that the system has for a problem or a pathology ..."

BA: "… the altered function of body systems, where, in my opinion, it can be placed in the context of adaptation, in the health of the patient as an adaptive capacity …"

BG: "... we can argue that we don't yet have the tools to measure it, that we don't yet have interoperator reliability ..."

LC: "There, it seems to me that we have treated something that we only palpate .... That is, as something that has to be treated because it has a meaning. We don't know if it has meaning!"

DFF: "... not being sure yet to detect something reliably and in a clinically meaningful way, then it creates space for further interpretations, enlargements and whatnot … there is the problem of reliability, there is also the problem of validity and so I say to myself: beyond, without wanting to trample on our perceptive self of which we have spoken before in abundance, but is there a common trace, a method, an operative way, which is more reliable than what we have now and which we have seen to be unreliable? Is there a diagnostic entity that we are all calling somatic dysfunction, that is a little bit more clinically relevant, a little bit more clinically valid than the one we are adopting now?"

TM: "... to the concept of somatic dysfunction certainly an initial clue is given to me by the tissue or tissue changes and then these tissue changes attract my attention ..."

BL: "... the less able the tissue is to adapt, the more dysfunctional the area."

PG:: "Personally I give a lot of importance to the provocation of the symptom, in addition to what has already been said, so I try in some way when a patient comes to stimulate the area that could be the source of the pain, so the manifestation of the symptoms for me assumes an important meaning because then it directs me to the region and the area to be treated."

LC: "... the osteopath finds things that I (patient) almost didn't know I had then access through intuition and palpation aspects that are not accessible to the body awareness of the patient at that moment ..."
DAG: " ... I have included a lot of the patient's perspective also in terms of where to start."
CG: " ... I'm only interested if the patient is also interested so I ask for feedback from the patient if he or she has an altered sensation from the palpatory point of view which can be of different nature: he or she perceives it harder, softer, painful, not painful, paresthetic, he or she perceives dysesthesias or hyperesthesias or other, so if an area is neurologically active for the patient ... if my palpation, if the tissue gives me information that is corroborated by the patient's sensation then at that point I consider that palpatory outcome clinically relevant. ... their (the osteopaths) hands find places that the patients didn't know they had as areas that were active and interesting from a somatic point of view and they discover thanks to palpation that they were interesting from a somatic point of view ... one of the possible roles of palpation is just this, to bring back to conscious levels of the patient some areas of the body that at that point he or she (the patient) is able to give a meaning to."

BG: "But the aspect to be considered is the aspect of semeiotics that we use which has a component of observation and a component of palpation and also a component of observation of the movement because the movement - we have all mentioned it - is fundamental in this kind of pathway. Then that we talk about permitted movement, that we talk about intrinsic movement, that we talk about movement in response to a solicitation from the operator or through breathing, whatever we want... but in the end, what we highlight is an alteration of mobility in the district that, locally, specifically is - in my opinion - expressiveness of altered function …"
BA: "... this motor variability is for me the clinical sign that I use in my clinical practice, ... I am always looking for that aspect, that is the free movement or the restricted moment, this variability is what I look for in my practice to look for what I consider altered in the patient and I need it to find, correcting it, ... "
VL: "... we see it a little bit differently, in the sense that for me palpation is, what I evaluate with palpation, is not so much a question of pressure but of movement. I palpate to analyse the movement, if we want to say it in simple terms, micro movements or as you know we have described a movement in the neutral zone. So, for me palpation is first of all to analyse how the system is moving, …"
DFF: "... I believe that in the parameter of the movement there is not only the articular aspect but, as BA said, there is also a whole system that we can investigate only because we induce a provocation that translates into movement ..."

VL: "... obviously when you talk about movement analysis you are including everything, skeletal aspects, myofascial, vascular, lymphatic, actually there is a lot in that movement and especially when we were talking about micro-movements [...] I'm trying to understand where that function has compromised and I use movement as a tool, a measure of adaptation ... the impaired function altered, ... I consider it an adaptation of the system to something that is required of it and this I assess through movement ..."
BG: "Not only a limitation of joint movement but also an inability to respond adaptively to, for example, an intrinsic test that can be breathing. If you ask a patient for a respiration and the area that you are palpating, not necessarily at the level of extrinsic movement, does not have a response, that area could have a significance of clinical relevance regardless of symptoms, alterations or pathology ..."
BL: "... the movement and the structure, the findings, the musculoskeletal part give me the weight of the dysfunction: the more the tissue is less able to adapt, the more dysfunctional is the area. But I start from an analysis of the movement and I start from the fact that we need to move to express ourselves, so if we are not able to express ourselves through movements of the soma, we will

hardly have a relationship life, the interface in my opinion is the soma and so I start from the analysis of the soma, from the analysis of the potentialities of the movement".

S3.3.1.2. Clinical Reasoning

TM: "... I (osteopath) relate all the different dysfunctions that I have been able to bring to light, then I relate them to the reason for the consultation and try to make a synthesis that is steeped in clinical reasoning and is based on my knowledge, experience and then I decide which and what to treat ..."
LC: ”... it is the diagnostic process and the therapeutic process, which is based on a shared decision-making process with the person, that tell me how to use palpation, in terms of type of touch, approach or technique …”
BG: “It could be added that in that case there was a specific diagnostic moment which must then be expanded, however, it must be completed by a much broader evaluation or relationship with the patient to be able to make a real osteopathic diagnosis, in an osteopathic clinic.”

S3.4.1. Sharing

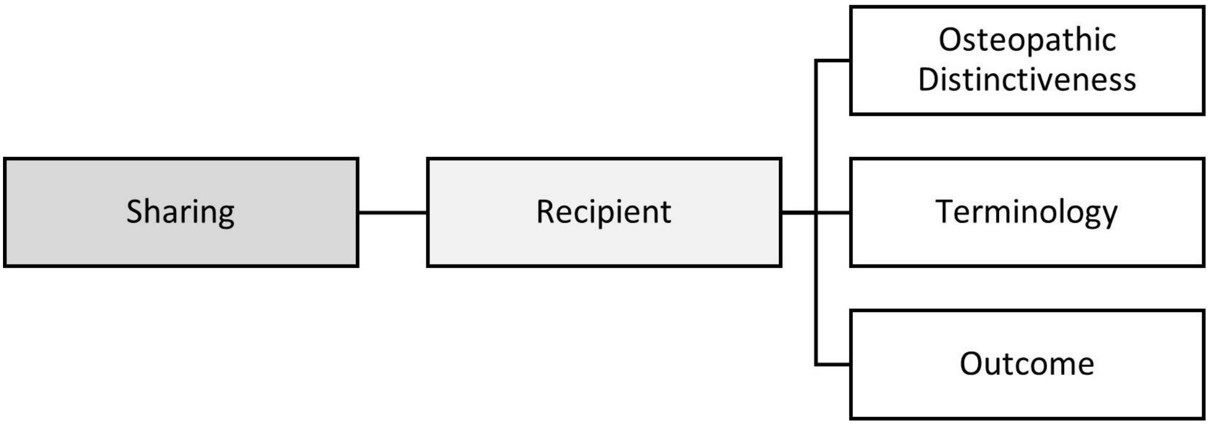

Figure S4. Categories and subcategories grouped by theme ‘Sharing’.

S3.4.1.1. Recipient

DAG: *“... the outcomes are all important because if I relate to myself some are significant, if I relate to the patient others are important and if I relate to the doctor others still.”*

LC: "... absolutely subjective internal dialogue and then I add a procedure that allows to explain to the world that subjective internal dialogue that has become a dialogue of the operator-patient relationship."
VL: ”... so the dialogue between osteopath and osteopath as well as the dialogue between osteopath and personal osteopath (themselves) ...”

S3.4.1.1.1. <u>Osteopathic distinctiveness</u>

VL: ”The information I share with the clinician comes from a specificity of my profession …”
BA: “What we value has a specific meaning for ourselves and that is difficult to communicate to other professionals. They don’t understand it.”
LC: “What is meaningful to me, is it meaningful to the patient? Is it meaningful to the rest of the world?”

S3.4.1.1.2. <u>Terminology</u>

BA: “… we (osteopaths) find it difficult to communicate with others because we use a terminology that others do not find in practice, in their studies and research [...]. Anterior iliac codifies a pelvic somatic dysfunction. For us it is a code that can justify an aspect but in the context of other professions they don’t understand it.”
CG: “The problem is terminology deficiency regarding many of the things that we (osteopaths) would like to be able to describe on the musculoskeletal system.”
VL: ”I can share with clinicians not strictly speaking with osteopathic terms.”

S3.4.1.1.3. <u>Outcome</u>

TM: “... through the measure we share.”
BL: “all of us have to face the international scientific world, based on scientifically measurable outcomes.”
DFF: “in my clinical practice I try to find a way to communicate with a clinician according to certain indicators”
