# Supplementary material for: Beliefs and Use of Palpatory Findings in Osteopathic Clinical Practice: A Qualitative Descriptive Study among Italian Osteopaths
Source: Healthcare (Basel). 2022 Aug 29;10(9):1647. doi: 10.3390/healthcare10091647 (PMC9498689; doi:10.3390/healthcare10091647)
Supplement: Supplementary file 1 [file healthcare-10-01647-s001.zip › Survey S1.pdf]

Initial anonymous survey administered to participants (n=12) prior to the start of the VFGs, the results of which gave an idea of the range of opinions in the group regarding PFs in osteopathic clinical practice and to initiate discussion in the fourth VFG:

PATIENT MANAGEMENT

How do you control the management of the patient with regard to assessment, treatment and prognosis? Describe it in brief:

1. Within the decision-making process, I insert moderators and process addressers. The addressers, consisting of the results of the objective examination of the reason for consultation, the functional objective examination of the body systems, the assessment of the patient's subjective comparable signs, allow the process to be guided towards a personalised and evidence-informed treatment. The moderators allow an assessment of the health status (allostatic index) and possibly the disease (validated scales for the presented disorder) with reference to the treatment.

2. The management of the patient in all the phases described above is linked to the use of assessment scales that allow the type of treatment and its dosage to be set. The patient's feedback, being the quickest assessment method even if not always the most reliable, represents the most used tool in my clinical practice.

3. Subjective patient report + somatic dysfunction/global neutrality.

4. Through anamnestic, palpatory and clinical semeiotic outcomes.

5. I manage the assessment and treatment phases by considering what emerges during the palpatory assessment in relation to what the patient has reported about his general health status, clinical history and traumatic events. In the prognosis there are many factors that come into play - in general, I manage it considering the two previous phases and the possible need to involve other professionals in the treatment pathway.

6. In general, I compile a file in which I note the relevant anamnestic data, the results of the manual assessment, the choice of the area to be treated, the technique used, the immediate response, any suggestions or requests for further diagnostic investigation. The same file is updated at each visit with a description of the symptoms (residual - changed or resolved), the results of any further diagnostic investigations and of the various assessments and treatments.

7. Assessment and re-evaluation through familiar symptoms (e.g. uncomfortable or painful daily activities), through comparable signs and through NRS. For treatment through "neurologically active" body areas/segments where there is agreement between the practitioner's perception/assessment and the patient's perception showing an influence on familiar symptoms or comparable signs. For prognosis, on the basis of the relevant scientific literature when present and on the empowerment of the patient's self-efficacy by trying to forage their ability to autonomously manage any changes in symptomatology.

8. I intend the Evaluation as a moment of synthesis between the patient's clinic and a specific osteopathic framework: the key element, which guides me most in this

passage, is the alteration of the quality of movement (always in relation to the clinic that the patient manifests). These are the same elements that I will then consider in the management of the treatment sessions for the definition of parameters such as frequency and therapeutic modalities. In my opinion, the prognosis must also be formulated on the basis of elements linked to the general health of the subject and his lifestyle (in a broad sense). I intend to build my clinical practice on the basis of my personal experience, without ever neglecting the comparison with EBM/EBP.

9. Through the analysis of subjective data (reported by the patient) and objective data with respect to the personalised therapeutic objective, the treatments administered and the expected outcomes on the basis of the patient's clinical condition. Pain scales, ROM, qualitative tissue variation/health status, assessment of activities of daily living-motor function, perception of patient satisfaction/expectations.

10. I rely on history, observation and clinical assessment. Based on good clinical practice, evidence and knowledge I orientate the prognosis.

11. Computerised clinical file including information related to the osteopathic evaluation and treatment, treatment progress, recording also anamnestic data and clinical-medical changes.

12. Through the information coming from the anamnesis, from the health professionals who have or have treated the patient, from my objective examination, from the subjective characteristics of the patient which influence the patient's reactions.

OUTCOMES IN OSTEOPATHY

Do you consider it necessary to use clinical/humanistic outcomes and related indicators for patient management?

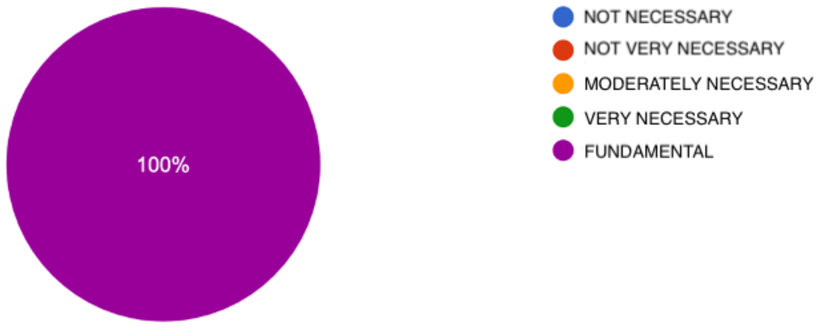

What types of clinical/humanistic outcomes do you think are most appropriate for osteopathic patient management? Multiple choice question.

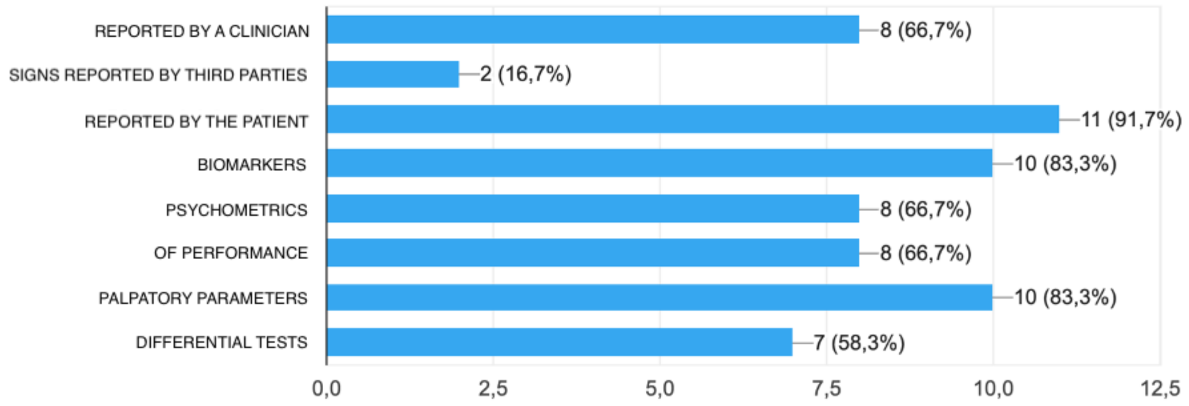

What clinical aspect do you consider important to monitor in the osteopathic management of the patient? Multiple choice question.

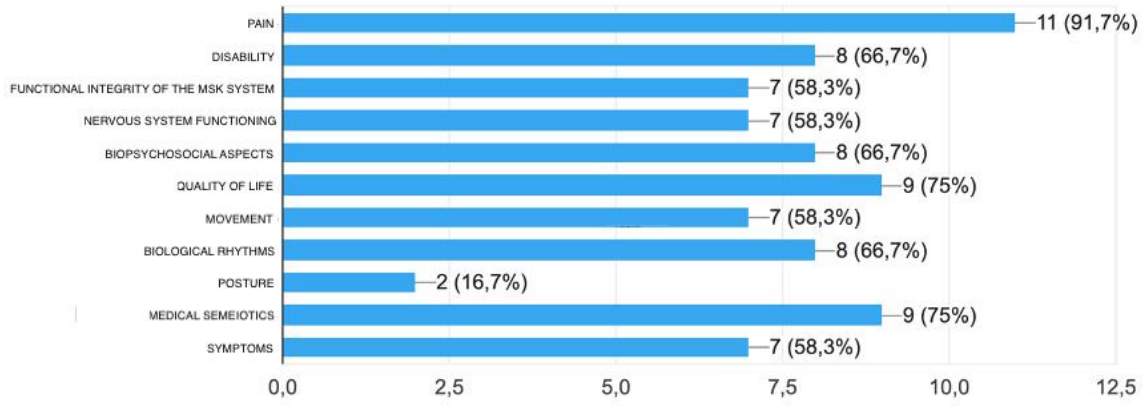

PALPATORY FINDINGS

What are the palpatory findings you use in patient management? Describe them in brief:

1. The palpatory outcomes used refer to tissue changes, positional and movement asymmetry, altered sensitivity. These parameters, characteristic of inflammatory processes, may be associated with a local adaptation syndrome and be detectable in an area referable to one or more anatomical structures (segmental dysfunction), to a region (somatic dysfunction); or they may be associated with a general adaptation syndrome and be detected in a generalised pattern in the body. In general, I attribute

clinical significance to palpatory outcomes only when provocation tests reveal their impact on patient responses.

2. Movement and pain provocation. Movement and its palpatory assessment is the most relevant clinical aspect in the osteopathic management of the patient. Pain and its provocation is the most reliable and repeatable aspect of manual therapy.

3. Somatic dysfunction assessed by means of TART criteria (mainly allowed movement, tissue quality and tenderness; less importance to asymmetry) + quality of the patient's global neutrality (within present movement).

4. Somatic dysfunction through Variability Model and thus movement asymmetry in the Neutral zone correlated with tenderness and texture.

5. Mobility test of the different body districts according to TART parameters, pain provocation test, respiratory assessment, cranial rhythmic impulse assessment.

6. Tissue alteration, range of motion, positional asymmetry.

7. DS through TART, but only if the operator's perception is corroborated by altered sensitivity/perception reported by the patient and only if they show an influence on familiar symptoms or comparable signs or if they are considered to be areas of interest by the patient.

8. Considering the reliability problems related to the palpatory examination, the first parameter in my opinion remains the altered variability of movement. This I believe should be assessed within the range of motion of a functional unit (hypothetical neutral zone) and not at the extremes. The alteration of tissue density and the asymmetries (especially of mobility) are in my opinion a consequence of the movement parameter. Sensitivity/discolouration may or may not be present in relation to possible sensitised states of the nervous system.

9. Clinical correlation of tissue properties/status (especially elasticity), freedom of movement in the rest position/neutral zone and biological movements/adaptability.

10. Painfulness, tissue resistance, movement reduction.

11. Pathogenetic palpation, associating tissue changes with organ functions in relation to SNA functions.

12. Body movement, which can be assessed mainly by using the musculoskeletal system and considering the relative asymmetry characteristics in the range around the resting position; soreness and finally tissue consistency.

Do you consider osteopathic palpatory findings to be part of diagnostic, outcome, prognostic evaluation?

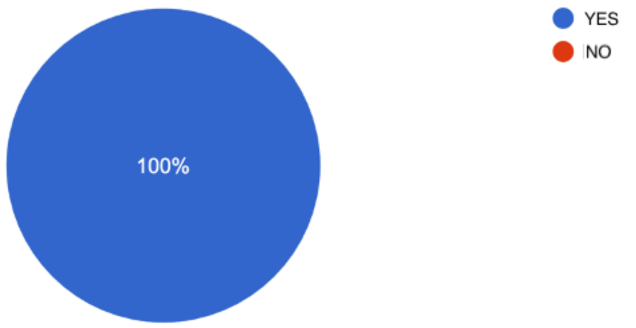

Do you think that the patient's clinical manifestations can be associated with palpatory findings?

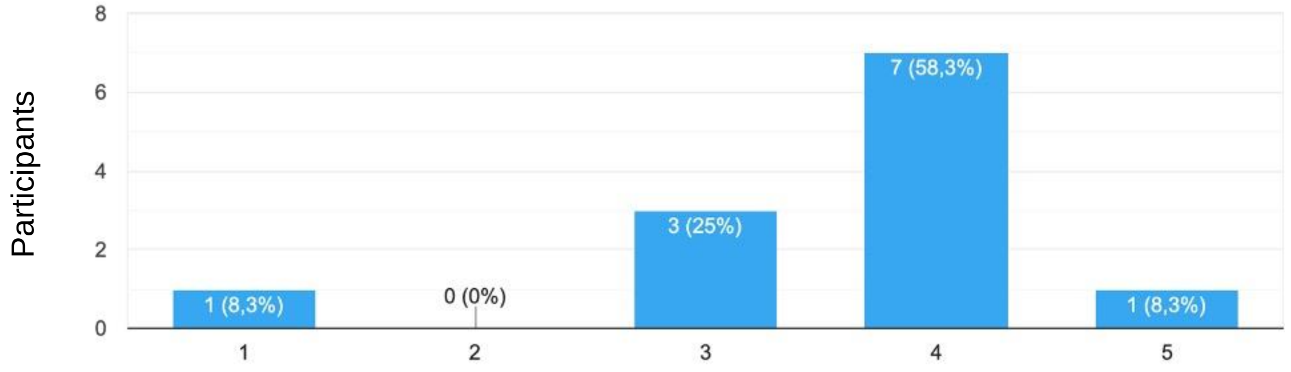

5-point numeric scale: 1 = few, 5 = a lot.

Do you consider the reliability of osteopathic palpatory findings important?

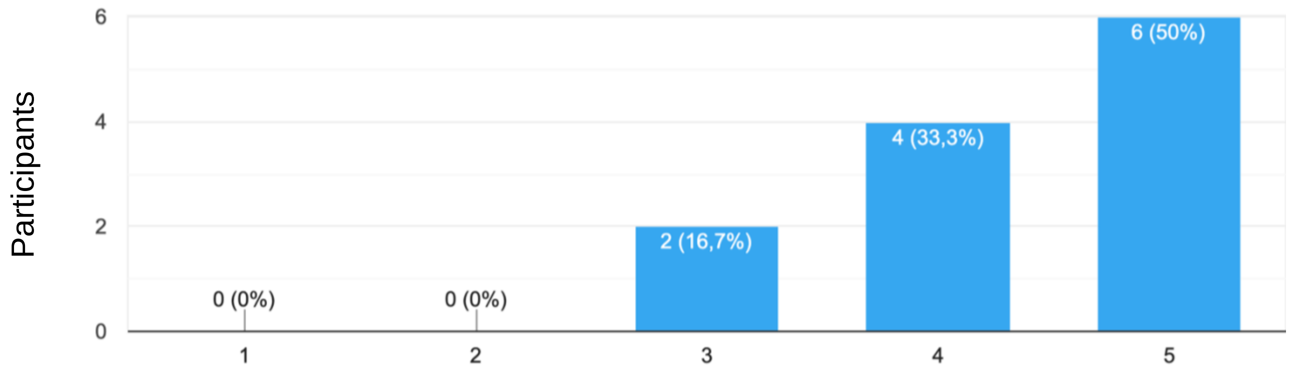

5-point numeric scale: 1 = few, 5 = a lot.

THOUGHTS

Taking into consideration some statements in Gary Fryer's 2017 article (Integrating osteopathic approaches based on biopsychosocial therapeutic mechanisms. Part 2: Clinical approach. Int J Osteopath Med. 26:36-43):
"For most people, a blend of biological and psychological factors will contribute to pain and dysfunction, and these factors should be addressed concurrently. In some people, some factors will predominate, and the emphasis of treatment will shift to address the relevant factors."

We ask participants to answer the next question:

In your clinical practice, how do you go about assessing the two factors (biological and psychosocial)? Describe it in brief:

1. Scales, validated questionnaires, objective examination to assess (monitor the trend) the patient's complaints and his adaptive capacity (allostatic index, objective functional examination of the biomechanical-postural, neurological, circulatory-respiratory, metabolic, psychosocial systems) are associated with the results of the osteopathic palpation.

2. The biological aspect is assessed by a careful anamnesis. The psychosocial aspect is a component that often emerges autonomously in the second or third session as a consequence of the therapist-patient relationship. Personally, working with a psychologist, we work as a team when the psychosocial aspect is predominant in the patient's disorder.

3. Biological: palpatory findings + evaluation of patients' verbal reports in relation to bodily functions (e.g. quality of alvus, skin manifestations) + instrumental examinations. BPS: (during dialogue) verbal reports of patients in relation to psychic symptoms (e.g., pain, emotional state).

4. I assess them mainly palpatory.

5. I assess psychosocial aspects with questions concerning the psychic sphere; biological factors are not assessed with biomarkers, I ask questions concerning the quality/quantity of sleep, the perception of tiredness/tiredness and lack of energy which, however, may be related to psychosocial factors.

6. The main aspect of this assessment is the dialogue with the patient and/or his family from which derives an analysis of needs and resources, a sharing of the treatment plan with the possible involvement of other professionals and with active participation of the patient. At the moment I do not use numerical scales for the evaluation of complex or chronic patients.

7. Biological factors through: anamnesis, eventual instrumental examinations, eventual haematochemical examinations, eventual medical diagnoses, tests aimed at differential diagnosis, osteopathic tests.

8. Psychosocial factors through: anamnesis, signs and symptoms of emotional dysregulation, investigation of the patient's beliefs about his disorder and analysis of

signs and symptoms of maladaptive beliefs, investigation of the socio-environmental context, any lifemarkers and psychomarkers, any blood tests, any medical diagnosis. It is necessary to consider both aspects, which will be fundamental in drawing up the assessment, treatment and prognosis. The fact remains that osteopathy must have its own specific and precise connotation/characterisation within the care professions, and its potential can only be limited to certain areas of intervention. The osteopath intervenes, through manual dexterity, on the structure of the body; he is able to act on the movement of the articular districts, on the metabolism of the tissues, on the vascular and neural functions and hypothetically on other aspects (neurological?, immune?, hormonal? psychological?) which could influence the general health of the subject. Therefore, the role towards the patient's psychosocial sphere can be one of integration with other professionals who, with more targeted skills, can better deal with the management of these aspects; it cannot and should certainly not have the pretension of replacing them.

9. Biological factor: objective and objective assessment of the cause of illness/reason for consultation. Qualitative/quantitative variation of dysfunctional parameters of structure and movement. Application of objective predictive/prognostic parameters where possible. Psychosocial factor: assessment of the emotional, cognitive and perceptual state reported by the patient. Analysis of the patient's and caregivers' behaviour (includes therapeutic education and behavioural ecology).

10. Using interview, observation and palpation.

11. Using validated tests, e.g. biomarkers and PROMs. In addition, the use of medical subject matter allows any biological and psychosocial correlations to be rationalised through a deductive method with medical semeiotics.

12. For both through the anamnesis, information from other professionals treating the patient, and the objective examination. In particular for the psychosocial one, some soft skills.
